# Contrasting phenotypes of putative proprioceptive and nociceptive trigeminal neurons innervating jaw muscle in rat

**DOI:** 10.1186/1744-8069-1-31

**Published:** 2005-10-24

**Authors:** Mark Connor, Ligia A Naves, Edwin W McCleskey

**Affiliations:** 1 Vollum Institute, Oregon Health & Sciences University, Portland, Oregon, USA; 2 Pain Management Research Institute, Kolling Institute, University of Sydney at Royal North Shore Hospital E25, St Leonards, NSW 2065, Australia; 3 Department of Physiology and Biophysic, Federal University of Minas Gerais, Belo Horizonte, Brazil

## Abstract

**Background:**

Despite the clinical significance of muscle pain, and the extensive investigation of the properties of muscle afferent fibers, there has been little study of the ion channels on sensory neurons that innervate muscle. In this study, we have fluorescently tagged sensory neurons that innervate the masseter muscle, which is unique because cell bodies for its muscle spindles are in a brainstem nucleus (mesencephalic nucleus of the 5^th ^cranial nerve, MeV) while all its other sensory afferents are in the trigeminal ganglion (TG). We examine the hypothesis that certain molecules proposed to be used selectively by nociceptors fail to express on muscle spindles afferents but appear on other afferents from the same muscle.

**Results:**

MeV muscle afferents perfectly fit expectations of cells with a non-nociceptive sensory modality: Opiates failed to inhibit calcium channel currents (*I*_Ca_) in 90% of MeV neurons, although *I*_Ca _were inhibited by GABA_B _receptor activation. All MeV afferents had brief (1 msec) action potentials driven solely by tetrodotoxin (TTX)-sensitive Na channels and no MeV afferent expressed either of three ion channels (TRPV1, P2X3, and ASIC3) thought to be transducers for nociceptive stimuli, although they did express other ATP and acid-sensing channels. Trigeminal masseter afferents were much more diverse. Virtually all of them expressed at least one, and often several, of the three putative nociceptive transducer channels, but the mix varied from cell to cell. Calcium currents in 80% of the neurons were measurably inhibited by μ-opioids, but the extent of inhibition varied greatly. Almost all TG masseter afferents expressed some TTX-insensitive sodium currents, but the amount compared to TTX sensitive sodium current varied, as did the duration of action potentials.

**Conclusion:**

Most masseter muscle afferents that are not muscle spindle afferents express molecules that are considered characteristic of nociceptors, but these putative muscle nociceptors are molecularly diverse. This heterogeneity may reflect the mixture of metabosensitive afferents which can also signal noxious stimuli and purely nociceptive afferents characteristic of muscle.

## Background

The masseter muscle is involved in many painful conditions which are grouped under the general heading of temporomandibular disorders [1]. Although a role of primary afferents innervating the masseter muscle in the development and maintenance of some of these pain states has been generally accepted, the cellular properties of masseter afferents have not been extensively investigated [2]. The trigeminal sensory system is unusual because the cell bodies of the trigeminal primary afferent neurons are located both in the trigeminal ganglion (TG) and in the mesencephalic nucleus of the 5^th ^cranial nerve (MeV) in the brainstem. The proprioceptive afferents located in the MeV include those arising from the masseter muscle [3], and this unique anatomical segregation enables direct comparison of the expression of proteins involved sensory transduction and its modulation between proprioceptive and other muscle afferents.

We have previously compared the types of P2X receptor and acid gated ion channel (ASIC)-mediated response between nociceptive and non-nociceptive sensory afferents [4,5], utilizing the MeV proprioceptors as the non-nociceptive neuronal population and either tooth pulp or cardiac afferents as the exemplar nociceptors. However, we have not previously compared the properties of different types of sensory afferent innervating the same tissue. We hypothesize that afferents from the masseter muscle that have different sensory functions will have different complements of signal detection and transduction molecules from each other, and that these different molecular profiles in part determine the response properties of the neurons to sensory stimuli. to In this study we have investigated the expression of sensory transduction-related ion channels and modulatory receptors in masseter afferent neurons located in the TG, which presumably represent a population of sensory neurons with mixed sensory modalities, and the proprioceptive masseter afferents located in the MeV. We determined the responses of these cell populations to the sensory mediators ATP, capsaicin and acid and potential analgesic agents such as μ-opioid agonists and baclofen [6,7]. There were significant differences between TG masseter afferents and MeV afferents in their voltage-gated sodium current (*I*_Na_) expression, responses to sensory mediators and μ opioid agonists.

## Results

We made recordings from 143 TG neurons and 31 brainstem MeV neurons labeled by injection of DiI into the masseter muscle. The diameter of the labeled ganglion cells ranged between 13 μm and 50 μm, the diameter of the cells isolated from the MeV nucleus ranged between 29 μm and 60 μm. We also recorded currents from 70 unlabeled TG neurons, with diameters between 12 μm and 50 μm.

### Action Potentials and Sodium Currents

**Figure 1:**
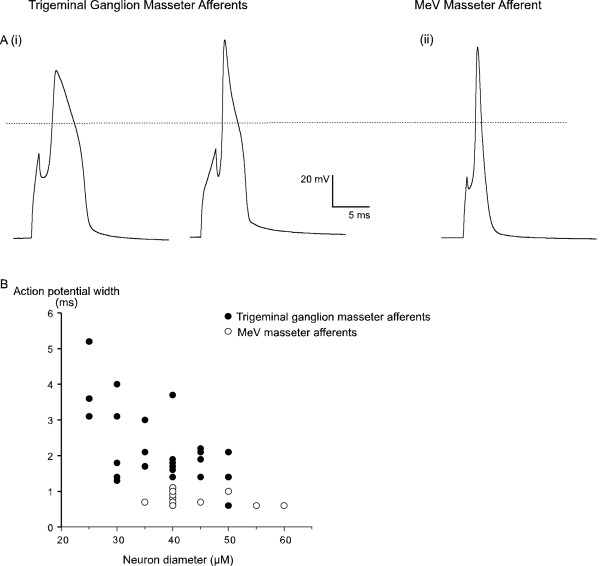
**Contrasting action potential characteristics of masseter muscle sensory afferents**. Action potentials were elicited by injecting a brief depolarizing current step to neurons held at -70 mV and the width measured at 0 mV. A(i) illustrates 2 example action potentials from trigeminal ganglion masseter afferents while A(ii) illustrates an action potential from a typical MeV nucleus masseter afferent. Action potentials widths are plotted against cell body diameter in B, note the wide range of action potentials widths in masseter afferents isolated from the trigeminal ganglion contrasting with the very tightly grouped action potentials widths from MeV masseter afferents.

Action potentials in masseter afferent neurons were examined in whole cell current clamp recordings (Figure 1). Neurons were held at -70 mV and sufficient current injected (0.5–3 ms duration) to elicit an action potential. Action potential duration was measured at 0 mV. Action potentials elicited from masseter afferents isolated from the TG varied considerably in width, with durations between 0.6 ms and 5.2 ms at 0 mV (average duration 2.2 ± 0.2 ms, n = 27, Figure 1B). By contrast, MeV masseter afferent action potentials were narrower and displayed much less variability with a mean AP duration of 0.8 ± 0.05 ms (range 0.6 ms to 1.1 ms, n = 13, Figure 1B).

**Figure 2:**
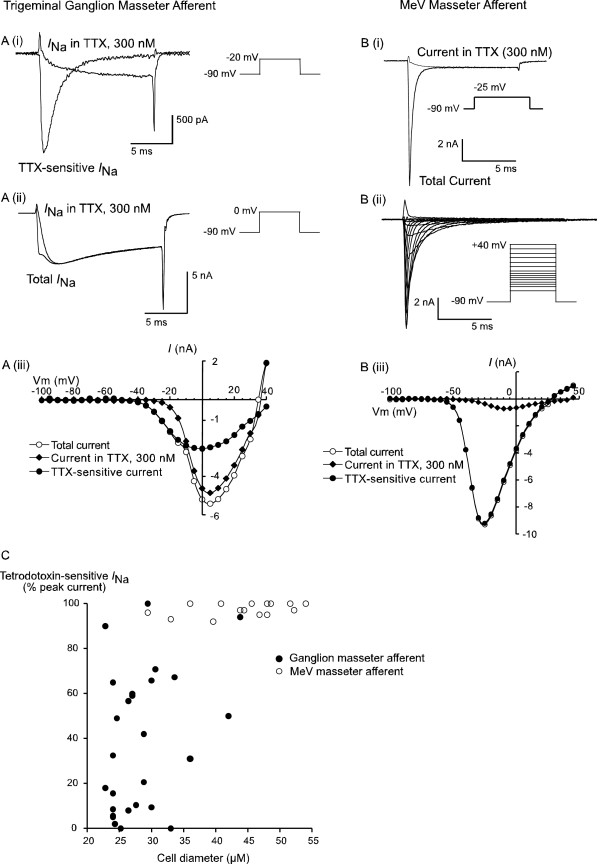
**Contrasting sodium currents in masseter afferents**. MeV masseter afferents express only TTX-sensitive *I*_Na_, but trigeminal ganglion masseter afferents display great variation in the proportion of TTX-sensitive to TTX-resistant *I*_Na _among cells. *I*_Na _were elicited by voltage steps from a holding potential of -90 mV, as diagrammed next to each set of traces. TTX-sensitive *I*_Na _were obtained by digitally subtracting the *I*_Na _remaining in TTX (300 nM) from the total *I*_Na_. A trigeminal ganglion masseter afferent is illustrated in A; (i) illustrates the TTX-sensitive and TTX-resistant *I*_Na _at -20 mV, a potential where TTX-sensitive current predominates while (ii) shows the total *I*_Na _and TTX-insensitive *I*_Na _at the potential where TTX-sensitive *I*_Na _is maximal. The peak amplitudes of the total *I*_Na_, TTX-sensitive and -resistant *I*_Na _are plotted in (iii). A typical MeV masseter afferent is illustrated in B, (i) illustrates the *I*_Na _and TTX-insensitive inward current at the test potential where *I*_Na _is maximal; (ii) shows the TTX-sensitive *I*_Na _over a range of test potentials. The peak amplitudes of the total *I*_Na_, TTX-sensitive and residual current (*I*_Ca_, see Table 1) are plotted in (iii). C) Illustrates the proportion of the peak inward current in TG and MeV masseter afferents that was sensitive to TTX (300 nM). The proportion of TTX-sensitive *I*_Na _for the cell illustrated in A) would be calculated at a test potential of +5 mV.

The types of voltage-dependent sodium channel currents (*I*_Na_) contributing to the action potentials in masseter afferents were examined using conventional voltage clamp recordings made in low extracellular Na (20 mM or 40 mM) in order to minimize the size of evoked *I*_Na_. The peak *I*_Na _was determined by stepping the neurons from a holding potential of -90 mV to potentials between -100 mV and +45 mV. Even in conditions of reduced external Na, the peak amplitude of evoked currents was substantial; peak *I*_Na _averaged 11 ± 3 nA in ganglion afferents (n = 27) and 22 ± 4 nA in masseter afferents isolated from the MeV nucleus (n = 15). Application of tetrodotoxin (TTX, 300 nM), a potent inhibitor of many types of *I*_Na_, inhibited the peak *I*_Na _in ganglion masseter afferents in a highly variable manner (Figure 2). The average inhibition by TTX was 38 ± 6% (n = 27) but the inhibition ranged in individual cells from nothing to 100%. In contrast, TTX completely inhibited the peak *I*_Na _in masseter afferents isolated from the MeV nucleus (99 ± 1%, n = 15, Figure 2).

### Modulation of *I*_Ca_

The calcium channel current (*I*_Ca_) density of neurons was determined by repetitively stepping the membrane potential from a holding potential of -90 mV to test potentials between -60 and +60 mV. The *I*_Ca _density of masseter afferents was similar to that of unlabeled cells (115 ± 6 pA/pF, n = 104 vs 109 ± 9 pA/pF, n = 53). The calcium channel density of masseter afferents isolated from the MeV nucleus was 36 ± 8 pA/pF (n = 12).

**Figure 3:**
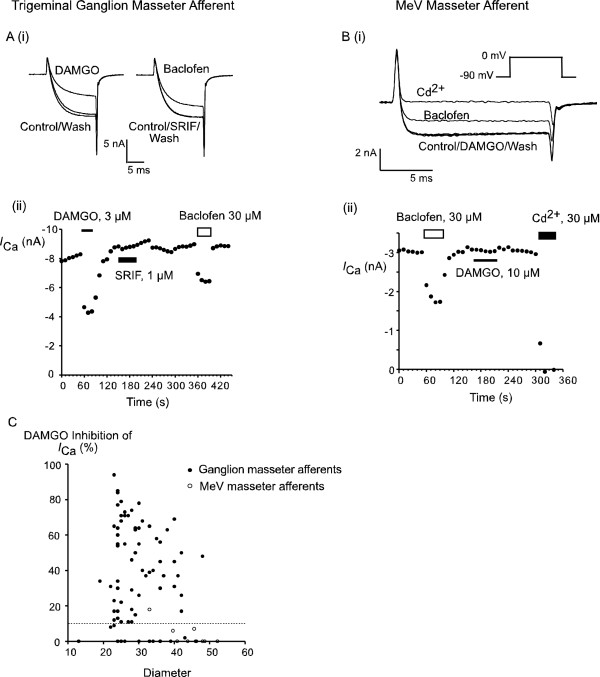
**Contrasting opioid modulation of masseter afferent *I*_Ca_**. *I*_Ca _were elicited by stepping from a holding potential of -90 mV to test potential of 0 mV. A) (i) Example *I*_Ca _traces from a trigeminal ganglion masseter afferent recorded in control conditions and in the presence of the μ-opioid agonist DAMGO and the GABA-B receptor agonist baclofen. (ii) A timeplot of the *I*_Ca _amplitude at 0 mV for the cell illustrated in (i). Drugs were applied for the duration of the bars. B) (i) Example *I*_Ca _traces from a MeV masseter afferent recorded in control conditions and in the presence of the μ-opioid agonist DAMGO the GABA-B receptor agonist baclofen and the *I*_Ca _blocker Cd^2+^. (ii) A timeplot of the *I*_Ca _amplitude at 0 mV for the cell illustrated in (i). Drugs were applied for the duration of the bars. C) The response of masseter afferents to a high concentration of DAMGO (3 or 10 μM) is plotted. A reversible inhibition of *I*_Ca _of 10% or greater was considered a significant response. Note that cells of all sizes responded to DAMGO, although only 1 MeV masseter afferent was DAMGO sensitive. All MeV masseter afferents were sensitive to baclofen.

The sensitivity of the masseter afferents to opioids was determined by examining the modulation of *I*_Ca _by agonists selective for μ-, δ- and κ-opioid receptors and the nociceptin receptor ORL1 (Figure 3). Cells were considered responsive if there was a reversible inhibition of the *I*_Ca _evoked by step from -90 mV to 0 mV of at least 10%. Maximally effective concentrations of the μ-opioid agonist DAMGO (3 μM–10 μM, [8]) inhibited *I*_Ca _in 74 of 92 labeled TG neurons (80%), and in 29 of 49 unlabeled neurons assessed at the same time (59%; P < 0.01, χ 2). In responsive masseter afferents DAMGO (3–10 μM) inhibited *I*_Ca _by 46 ± 3%, in responsive unlabeled cells the inhibition of *I*_Ca _was 39 ± 4%. We have previously arbitrarily divided trigeminal neurons into small (diameter <30 μm), intermediate (diameter between 30 μm–40 μm) and large (diameter >40 μm) cells [8]. DAMGO inhibited *I*_Ca _in 85% of small masseter afferents, in 73% of intermediate diameter cells and in 82% of large cells (Figure 3C). The inhibition of *I*_Ca _by DAMGO was 48 ± 4% in small masseter afferents, 50 ± 4% in intermediate cells and 36 ± 4% in large masseter afferents. Neither the κ-opioid agonist U69-593 (3 μM, n = 10) or the δ-opioid agonist deltorphin II (n = 13) inhibited *I*_Ca _in masseter afferents. U69-593 inhibited *I*_Ca _in 1 of 11 unlabeled cells tested (by 57%), deltorphin II did not inhibit *I*_Ca _in any unlabeled cells (n = 11). The ORL1 agonist nociceptin (300 nM-1 μM) inhibited *I*_Ca _in 6/15 (40%) of masseter afferents (inhibition of *I*_Ca _was 41 ± 9%) and in 5/12 (42%) of unlabeled cells (inhibition of *I*_Ca _was 38 ± 12%). The GABA_B _receptor agonist baclofen inhibited *I*_Ca _in most ganglion masseter afferents (31/35; 89%, inhibition of *I*_Ca _was 26 ± 4%) and unlabeled neurons (15/20; 75%, inhibition of *I*_Ca _was 24 ± 4%).

We have previously reported that somatostatin receptors preferentially inhibit *I*_Ca _in large tooth pulp nociceptors when compared with small nociceptors [8]. Somatostatin inhibited *I*_Ca _in 11/39 labelled masseter afferents and 3/15 unlabelled afferents. Somatostatin inhibited *I*_Ca _in 5/19 small masseter afferents and 3/7 large masseter afferents (P = 0.4, χ2). The vast majority of masseter afferents that did (8/11) or did not (22/28) respond to SRIF also responded to DAMGO.

DAMGO (3 μM) inhibited *I*_Ca _in only 1/11 MeV masseter afferents examined (by 18%). Baclofen (30 μM–100 μM) inhibited *I*_Ca _in all MeV masseter afferents examined, by an average of 33 ± 2% (n = 10, Figure 3B).

### Nociceptive Channels

#### TRPV1

The sensitivity of trigeminal neurons to the TRPV1 agonist capsaicin (3 μM) was determined by superfusing capsaicin onto neurons voltage clamped at -70 mV. Neurons with reversible capsaicin-induced currents of greater than 500 pA were considered to be sensitive. Many masseter afferents (65/137; 47%) and most unlabeled neurons (37/62; 60%) responded to capsaicin, with inward currents of up to 30 nA. Capsaicin produced inward currents in cells of all diameters, from 12 μm to 50 μm. Masseter afferents isolated from the MeV nucleus did not respond to capsaicin (n = 8).

#### ATP

The sensitivity of trigeminal neurons to ATP (50 μM) was determined by a 500 ms application of neurotransmitter to neurons voltage clamped at -70 mV. Neurons with reversible ATP-induced currents of greater than 500 pA were considered to be sensitive. Most masseter afferents (40/60; 66%) and unlabeled neurons (12/17; 70%) isolated from the TG were sensitive to ATP, with currents of up to 9500 pA. Masseter afferent responses to by ATP application were kinetically diverse, with many cells displaying a rapidly activating current that was more than 90% inactivated after 500 ms (19/40; 48%), or a mixture of rapidly activating and sustained currents (15/40; 38%). Some cells displayed slowly activating currents that did not significantly inactivate at the end of the 500 ms pulse (6/40; 15%). Masseter afferents isolated from the MeV displayed small sustained currents in response to ATP application (average amplitude of 150 ± 36 pA, n = 8).

#### Acid

**Figure 4:**
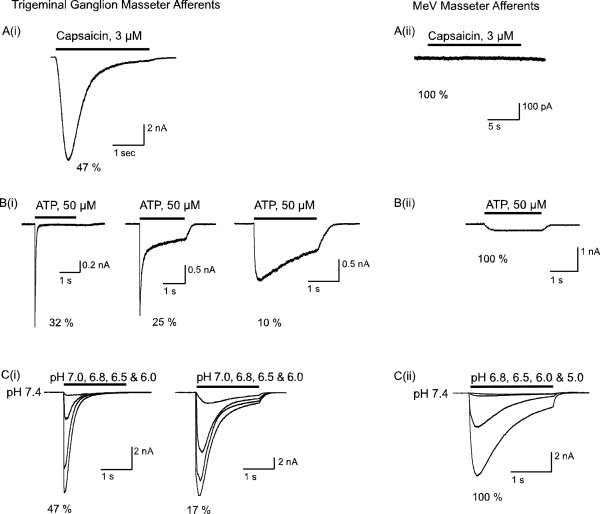
**Contrasting masseter afferent responses to nociceptive mediators**. In these experiments cells were voltage clamped at -70 mV and mediators applied for the duration of the bars. The percentage of all tested cells that responded with currents similar to those illustrated is shown below each example trace. A) Perfusion of the TRPV1 agonist capsaicin produced an inward current in many (i) trigeminal ganglion masseter afferents but no (ii) MeV masseter afferents. B) Perfusion of ATP produced inward currents in most (i) trigeminal ganglion masseter afferents and all (ii) MeV masseter afferents. The ATP currents in ganglion afferents exhibited a variety of kinetic profiles while those elicited from MeV neurons were uniformly small and non-desensitizing. C) Changing the pH of the perfusion solution from 7.4 to various more acidic values produced large inward currents in most (i) trigeminal ganglion masseter afferents and all (ii) MeV masseter afferents. Note that MeV neurons required greater changes in pH (7.4 to 6.5) to produce detectable inward currents than TG neurons (7.4 to 7.0). Acid-induced currents in TG neurons most commonly inactivated completely within about 500 ms, but in about 30% of cells a substantial inward current remained at the end of the 2 sec acid perfusion to pH 6.0.

The sensitivity of masseter afferents to extracellular acidification was assessed by varying the pH of the external solution from pH 7.4 to pHs between 7.0 and 5.0. Neurons were voltage clamped at -70 mV and acid solutions perfused for 5 s. Cells with an inward current of at least 500 pA for a given pH were considered sensitive. Most masseter afferents (45/70; 64%) displayed robust inward currents when the extracellular pH was changed from 7.4 to 6.8 (average amplitude 4.9 ± 0.5 nA). Similarly, changing extracellular pH from 7.4 to 6.8 produced large inward currents in most unlabeled ganglion afferents (11/19 cells (58%), average amplitude 6.9 ± 1.4 nA). The inward currents elicited by changing pH in ganglion masseter afferents could be distinguished by the degree of inactivation of the current during a pH application to pH 6.0 (Figure 4). The peak inward current in response to a pH step from 7.4 to 6.0 was 11.8 ± 1.5 nA, which declined to 1.2 ± 0.3 nA by 1.5 s (n = 45). In 33/45 (73%) of cells the inward current at 1.5 s was less than 10% of the peak (average sustained current 3 ± 0.5% of peak), in 12/45 (27%) of cells the sustained current was greater than 10% of the peak (average sustained current 24 ± 4% of peak).

In masseter afferents isolated from the MeV nucleus changing extracellular pH from 7.4 to 6.8 produce an inward current of only 120 ± 25 pA (n = 11). However, changing the pH from 7.4 to 6.0 produced inward currents of 3.5 ± 1 nA in MeV cells, and in 3 cells further decreasing the pH to 5.0 produced even larger inward currents (7.5 ± 0.3 nA).

### Co-expression of Nociceptive Channels

**Figure 5:**
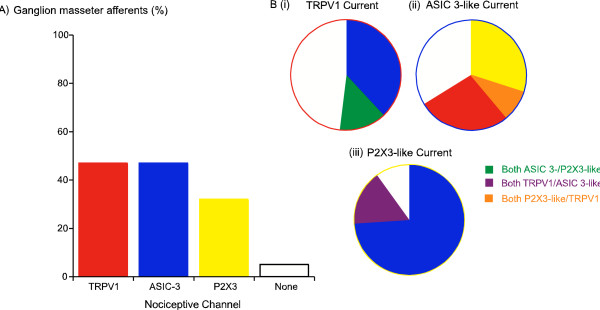
**Prevalence and co-expression of putative markers of nociceptive sensory neurons in trigeminal ganglion masseter afferents**. A) The proportion of masseter afferents from TG that expressed substantial (> 500 pA) currents mediated by TRPV1-, ASIC3- and P2X3-containing channels is plotted. Virtually all ganglion masseter afferents expressed at least one of these channels. B) Co-expression of nociceptive channels in cells expressing (i) TRPV1-like currents, (ii) ASIC-3 like currents and (iii) P2X_3_-like currents. TRPV1 expressing cells often expressed ASIC3-like currents, and P2X_3_-like currents were only found in the cells also expressing ASIC3-like currents. Cells with ASIC3-like currents often expressed either TRPV1 or P2X_3_-like currents and occasionally both together. Cells with P2X_3_-like currents usually expressed ASIC3-like currents but TRPV1-like currents were only found in the population of these cells also expressing ASIC3-like currents. The numbers of cells in each population can be found in the Results.

We were able to examine the co-expression of all 3 of the putative nociception-related ion channels in 55 TG masseter afferents (Figure 5). Only 4/55 (7%) of neurons failed to express one of either TRPV1, ASIC or P2X_3_-like current (Figure 5a). Conversely, only 3/55 (5%) of cells expressed all 3 types of current. About half the neurons (29/55, 53%) expressed at least 2 of the channels while the remainder (22/55, 40%) expressed only one of the nociception-related channels. The data for co-expression of channels is illustrated in Figure 5b. ASIC3-like currents were the most commonly co-expressed channel in neurons expressing TRPV1 or P2X_3_-like currents, while no neurons expressed only TRPV1 and P2X_3_-like currents.

## Discussion

Recordings from muscle afferent fibers have provided a wealth of information about the response properties of these cells in physiological and pathophysiological situations, and how these responses are modified by sensory mediators [9]. However, ion channel activation and the signal transduction cascades modulating primary afferent excitability are most directly studied by making electrophysiological or optical recordings from sensory neuron cell bodies, and the present study provides some of the first descriptions of the electrophysiological properties of isolated sensory neurons innervating muscle. The results highlight the differences in the molecular signatures of proprioceptive muscle afferents and other muscle afferents, as well as the differences between muscle afferents and those which innervate other structures in the head such as teeth [4,8].

Sensory neuron modality can only be determined in *in vivo *or intact *ex vivo *preparations, thus we cannot assign a definitive physiological function to the cells in the present study. However, sensory neurons that detect potentially noxious stimuli (nociceptors) are thought to preferentially express a number of ion channels not normally found in other primary afferents. For example, expression of the TTX-resistant sodium channels Na_V_1.8 and Na_V_1.9 has been strongly correlated with a nociceptive sensory modality in *in vivo *recordings made from sensory neuron cell bodies [10,11]. Channels such as the vanilloid receptor TRPV1 are thought to be expressed exclusively by nociceptors because they are normally activated by demonstrably noxious stimuli and there is a strong correlation between selective pharmacological activation of the channels and human sensations [7,12]. Other channels are thought to be associated with nociceptors because their biophysical properties are sufficient to explain a response to a noxious stimulus by a subset of sensory neurons. For example, ASIC3 channels are activated by the modest changes in extracellular calcium and pH that accompany cardiac ischaemia and are highly expressed in a subset of cardiac sensory afferents that are presumed to transmit the pain of angina [5]. The assignment of ion channels to nociceptive neurons has also been made based on correlating channel expression with other putative markers of nociceptors including small soma diameter, expression of substance P, calcitonin gene related peptide or TRPV1 and expression in sensory neurons projecting to tissues from which the only conscious sensation is pain [4].

Almost all the masseter afferents isolated from the TG expressed significant amounts of at least one of the putative "nociceptive" ion channels we examined in this study; capsaicin activated TRPV1 channels, acid activated ASIC-3-like channels or ATP activated P2X_3_-like channels. With the exception of cells expressing TRPV1, a nociceptive phenotype cannot be reasonably inferred from the expression of any one channel, however significant numbers of masseter afferents expressed two or more of the channels we examined. Almost all cells expressing P2X_3_-like channels also expressed ASIC3-like currents and about 25% also expressed TRPV1; most cells expressing ASIC3-like currents also expressed either P2X_3_-like currents or TRPV1, and more than 50% of the TRPV1 expressing cells also expressed either P2X_3_-like currents or ASIC3-like currents. These data indicate that most TG masseter afferents can detect a noxious stimulus, but whether this represents their primary or only function remains unknown. By contrast, masseter afferents isolated from the MeV nucleus did not express TRPV1, P2X_3_-like or ASIC3-like channels, consistent with their function as purely proprioceptive muscle spindle afferents [3].

There is considerable evidence that a proportion of muscle afferents can reliably signal stimuli in both the innocuous and noxious range, and the properties of some of these afferents are consistent with the expression patterns of the channels in muscle afferents found in the present study [9]. In particular, afferents that are activated by the changing metabolic state of muscle (metaboreceptors) [13] appear to express channels classically associated with nociceptors, such as TRPV1, but clearly signal non-noxious information as well. Thus, lactic acid stimulation of muscle afferents in rat produces a classic cardiovascular pressor response that is sensitive to the ASIC channel antagonist amiloride but not to the TRPV1 antagonist capsazepine [14]. However, the lactic acid-induced response is attenuated after pretretament with the potent TRPV1 agonist resiniferatoxin, which desensitizes or destroys TRPV1-expressing nerves [14]. Further, while capsaicin produces a pressor response, blocking TRPV1 does not inhibit a contraction-induced pressor response [15]. Recordings from muscle afferents also show that capsaicin activates a population of Group III and Group IV afferents, some of which also proton-sensitive [16]. Thus it seems that a significant proportion of muscle afferents involved in producing activity-induced cardiovascular reflexes, perhaps mediated by activation of ASIC channels, also express TRPV1. Injection of capsaicin into human masseter muscle is painful [17], so there is no question that there are TRPV1 expressing afferents in muscle that transduce noxious stimuli. Our findings that about 50% of TRPV1 containing masseter afferents also expressed ASIC channels, and 30% of ASIC expressing afferents expressed TRPV1 are consistent with these results. There is no other information about the co-expression of TRPV1, ASIC and P2X receptors in afferents from the masseter muscle, although a relatively limited co-expression of TRPV1 and P2X_3 _receptors has been reported in gastrocnemius-soleus muscle afferents [16].

The ASIC channels in trigeminal masseter afferents seemed to be largely comprised of homomeric ASIC3 channels or ASIC channel heteromers containing ASIC3. ASIC3 channels are highly sensitive to changes in extracellular pH and lactate and if activated by a substantial change in pH they desensitize significantly more rapidly than other ASIC channels [5,18,19]. The pH 6-induced currents in most trigeminal ganglion masseter afferents desensitized more than 90% during the 1.5 s proton application, while in the remaining neurons the significant residual current (> 10% of the peak) suggested the presence of other ASIC subunits in these cells, probably ASIC1 [18]. Thus, the majority of ASIC currents observed in TG ganglion afferents had similar properties to those found in rat cardiac afferents (18), which are thought to be ASIC3-mediated. However, in the absence of selective blockers of ASIC subunits, we cannot definitively assign the currents we observed to specific ASIC subunits or combinations of subunits. MeV masseter afferents exhibited robust acid-induced currents but these were less sensitive to changes in extracellular pH and desensitized much more slowly than ganglion neuron ASIC currents.

In the only previous study of ASIC channel function in muscle afferents, 50% of sensory neurons labeled from the gastrocnemius muscle responded to pH 5.0 solution with robust inward currents [20]. The currents elicited in 30% of the cells were tentatively assigned to ASIC3/ASIC2b heteromers. The crucial role of ASIC3 channels in muscle-associated sensory function is underlined by the main finding of that study, which is that ASIC3 channel expression is required for the long lasting hyperalgesia produced by repeated acid injection in muscle [20]. The currents we observed in the majority of rat masseter afferents differ from those reported in mouse dorsal root ganglion neurons [21], primarily due to the lack of a significant sustained current component at pH 6.0-in our experiments this component was only 3% of the peak current. However, our conclusion that ASIC3 forms an essential part of masseter afferent ASIC channel complexes, is similar to that reached by others based on experiments in mouse DRG neurons from ASIC-null mice [21].

### Action Potentials and Sodium Channels in Masseter Afferents

The action potentials of the MeV masseter afferents were narrow and lacked an inflection on the downward component of the current, consistent with previous recordings from acutely isolated MeV neurons [22] and MeV neurons in brain slices [23,24]. The *I*_Na _recorded from MeV masseter afferents were completely blocked by TTX. These data are consistent with reports that muscle spindle afferents do not express detectable Na_V_1.8 or Na_V_1.9 immunoreactivity [10,11]. The narrow action potentials of proprioceptive afferents are consistent with the very rapid firing rates that these neurons achieve-exceeding 200 Hz (e.g. [25]). By contrast, the masseter afferents isolated from the TG had a wide range of action potential widths and shapes, and most cells had a significant component of TTX-resistant *I*_Na_. TTX-resistant *I*_Na _are subject to acute regulation by a variety sensory mediators acting via G protein-coupled or tyrosine kinase-linked receptors, particularly prostaglandins, bradykinin and nerve growth factor [26]. The changes in *I*_Na _availability produced by these mediators mean that afferents expressing TTX-resistant *I*_Na _are likely to be subject to rapid changes in excitability reflecting the state of the tissue they innervate. Wider action potentials and greater amounts of TTX-resistant *I*_Na _are strongly correlated with a nociceptive modality, but these properties vary between afferents of different conduction velocity classes as well as between afferents of different modality within a class [27], and one cannot define a neuron as nociceptive simply on the basis of a action potential duration or its sensitivity to TTX. Nevertheless, within the TG masseter afferents, which presumably contain cells with a nociceptive function, smaller neurons tended to have wider action potentials. There was no such relationship apparent within the proprioceptive MeV masseter afferents.

### P2X Receptors

We found a wide variety of ATP-induced currents in TG masseter afferents, similar to results from other studies in sensory neurons [28-30]. Messenger RNA and receptor-like immunoreactivity for 6 of the 7 cloned subtypes of P2X receptor are found in the trigeminal ganglion [31,32] and the currents we recorded are likely to be comprised of a mixture of homo- and heteromeric P2X receptor channels. Although attempting to define the P2X subunits responsible for the variety of ATP currents was beyond the scope of this study (but see [28]) we attributed the rapidly desensitizing ATP current observed in some masseter afferents to P2X_3 _receptor activation. Rapidly desensitizing ATP currents in sensory neurons have been reported to depend on the presence of the P2X_3 _gene or have been identified pharmacologically as P2X_3 _receptors [33-35] and although the kinetically similar P2X_1 _receptor has been shown to be present in sensory neurons by immunohistochemical methods, there is little electrophysiological evidence for currents mediated by P2X_1 _receptors in rat or mouse sensory neurons [4,29].

P2X_3 _subunits make a major contribution to the ATP currents in trigeminal neurons projecting to the tooth pulp, both as P2X_3 _receptor homomers and putative P2X_2_/P2X_3 _heteromers [4]. Relatively fewer masseter afferents express P2X_3_-like immunoreactivity [36]. In the present study we observed rapidly desensitizing ATP currents either alone or in combination with other P2X currents in about 55% of TG masseter afferents, which is similar to the proportion of tooth pulp nociceptors which displayed fast ATP currents (44%, [4]). P2X_3_-like immunoreactivity has been reported in about 25% of masseter afferents [36], which is a considerably smaller proportion than suggested by the present report. These differing results may reflect differing sensitivities of immunohistochemistry and electrophysiology, they may be due to the presence of some P2X_1_-containing currents in masseter afferents or perhaps arise from the short time the isolated neurons spend in culture. 30 – 40% of tooth pulp afferents can be labeled with P2X_3 _antiserum [4,37] but it is interesting to note that more than 50% of tooth pulp afferents challenged with ATP also displayed persistent currents. This indicates that while P2X_3_-containing ATP receptors are found in many putative nociceptors, they are not the only P2X subunits that may detect noxious stimuli signalled by ATP.

### Opioid Modulation of Calcium Channels

The relatively high μ-opioid receptor sensitivity of jaw muscle afferents is similar to that reported in afferents projecting to hindlimb muscles, where about 75% of cells were sensitive to DAMGO [38]. The apparently high sensitivity of muscle afferent *I*_Ca _to μ-opioid agonists contrasts with the reported low frequency of μ-opioid agonist modulation of *I*_Ca _in skin and colonic afferents (approximately 10%, [39]). The relative insensitivity of MeV *I*_Ca _to DAMGO (1 of 11 cells responding) is consistent with the extremely low mRNA abundance in these cells (2 of 72, [40]). The modulation of *I*_Ca _in MeV neurons by the GABA_B _receptor agonist baclofen is in contrast to the lack of affect of baclofen on the membrane properties of MeV neurons in slices [41].

Interestingly, the μ-opioid receptor sensitivity of masseter muscle afferents differs markedly from that reported for the "purely nociceptive" afferents from tooth pulp [8,40]. DAMGO inhibited *I*_Ca _in more masseter afferents than tooth pulp afferents, regardless of cell size (80% versus 42% respectively [40]), and most strikingly, DAMGO was equally effective in small and large masseter afferents (85% and 82% of cells inhibited respectively). By contrast, DAMGO inhibited *I*_Ca _in only 30% of large tooth pulp afferents [8]. As 30% of large masseter afferents had substantial capsaicin currents (> 500 pA), indicating that these cells are likely to be nociceptors, our data suggest that μ-opioid receptors may be differentially expressed in distinct populations of nociceptors projecting to different tissues in the head, i.e. preferentially expressed in muscle nociceptors versus tooth pulp nociceptors. These data suggest that opioid analgesics may be better at relieving some types of head pain than others, and further that the endogenous opioid analgesic systems of the periphery may display differential effectiveness against nociceptive stimuli arising from distinct structures. The apparently high expression of opioid receptors in TG masseter afferents also suggests that these receptors may have functions in muscle physiology other than simple inhibition of nociception.

## Conclusion

This study demonstrates that most masseter muscle afferents isolated from the trigeminal ganglion express one or more ion channels associated with detecting noxious stimuli, while masseter muscle proprioceptive afferents isolated from the MeV a different array of ion channels consistent with their non-nociceptive phenotype. It remains to be seen whether this profile is typical of sensory innervation of skeletal muscle, and whether the phenotypes described in this study undergo significant changes in chronic pathologies of the masseter muscle or associated nerves.

## Methods

### Cell labelling

All experiments were carried out using protocols approved by the OHSU Institutional Animal Care and Use Committee. Masseter afferents were labeled as outlined in detail in [42]. Briefly, male Sprague-Dawley rats between 5–8 weeks old were anesthetized with a s.c. injection of "rat cocktail" consisting of ketamine (55 mg kg ^-1^), xylazine (5.5 mg kg ^-1 ^and acepromazine (1.1 mg kg ^-1^). A small incision was made in skin overlying each masseter muscle and 5 × 1 μl injections of DiI (5% in DMSO) were made into each muscle with a Hamilton 10 μl syringe. The wound was closed with cyanoacrylate glue and the animals returned to their cages. Sensory neurons were isolated 2 weeks after surgery.

### Cell Isolation

Cells were isolated from trigeminal ganglia essentially as described in [42]. Briefly, rats were anaesthetized with halothane (4%), and killed by decapitation. The trigeminal ganglia were removed and placed in cold Ca^2+^/Mg^2+^-Free Hanks Solution (CMF Hanks). Ganglia were cut up with iridectomy scissors and incubated at 35°C for 20 minutes in CMF Hanks plus papain (20 U ml^-1^), followed by 20 minutes in CMF Hanks plus dispase (4 mg ml^-1^) and collagenase (3 mg ml^-1^). The enzyme incubation was stopped with F-12 media supplemented with 10% fetal bovine serum (FBS), 50 U/ml penicillin/streptomycin, and the cells were released by gentle trituration through decreasing bore Pasteur pipettes with fire-polished tips. Cells were plated on plastic culture dishes precoated with poly-D-lysine and laminin. After the cells had settled they were cultured in a humidified chamber at room temperature in Leibovitz's L-15 medium supplemented with 10% FBS, 50 ng ml^-1 ^NGF, 5 mM glucose, 5 mM NaHEPES and 50 U ml^-1 ^penicillin/streptomycin.

Cells were isolated from the MeV nucleus by a modification of the methods outlined in [42]. Briefly, rats were anaesthetized with halothane and killed by a blow to the chest. The brain was rapidly removed and a block containing the brainstem immersed in ice cold artificial cerebrospinal fluid of composition (mM): NaCl 126, KCl 2.5, NaH_2_PO_4 _1.4, MgCl_2 _1.2, CaCl_2 _2.4, glucose 11, NaHCO_3 _25. Slices of the brainstem (400 μM) containing the MeV region were made with a Leica vibratome and the region containing the MeV cells subdissected with fine needles. The tissue chunks were placed in modified HBS (Solution 1 containing 10 mM MgCl_2_, 2 mM CaCl_2_) containing papain, 20 U ml^-1 ^and incubated for 3–5 minutes at 37°C. The enzyme incubation was stopped with F-12 media supplemented with 10% FBS, 25 ng ml ^-1 ^NT-3 and 50 U/ml penicillin/streptomycin, and the cells were released by gentle trituration through decreasing bore Pasteur pipettes with fire-polished tips. The cells were plated onto culture plates with confluent, quiescent glia and cultured overnight at 37°C.

### Electrophysiological Recording

Ionic currents from trigeminal neurons were recorded in the whole-cell configuration of the patch-clamp method [43] at room temperature (22–24°C), as described in [44]. The solutions used to record different types of current are listed in Table 1. Dishes were continually perfused with HEPES buffered saline (HBS, Solution 1). Calcium channel currents (*I*_Ca_), were recorded in Solution 2, recordings of sodium channel currents (*I*_Na_) were made in Solution 3. Recordings of *I*_Ca _and *I*_Na _were made with fire polished borosilicate pipettes (A-M Systems #603500, Carlsborg WA) filled with (in mM): CsCl 120, MgATP 5, NaCl 5, Na_2_GTP 0.3, EGTA 10, CaCl_2 _2 and HEPES 20, pH 7.3, resistance approximately 2 MΩ. In the cells where *I*_Ca _and *I*_Na _were recorded, capsaicin currents were also recorded with the above internal solution. Action potentials were recorded in Solution 4, with an internal solution that consisted of (mM): K methanesulphonate 115, KCl 5, NaCl, 8, MgCl_2_, 1, MOPS 10, MgATP 2, Na_2_GTP 0.3, BAPTA-K_4 _10, pH 7.0. In these recordings thin walled 7052 type glass (Garner Glass Company, Claremont CA) was used.

**Table 1:** External Solutions

	Solution 1 HBS	Solution 2 *I*_Ca _buffer	Solution 3 *I*_Na _buffer	Solution 4 AP buffer*
NaCl (mM)	140		20 or 40	145
KCl (mM)	2.5			5
CaCl_2 _(mM)	2.5	2.5	1	2
MgCl_2 _(mM)	1	1	3	1
HEPES (mM)	10	10	10	10
MES (mM)				10
TEA (mM)		140	120 or 100	
CsCl (mM)		5		

All external solutions contained glucose (*5 mM or 10 mM) and the pH was adjusted to 7.4 with either NaOH or CsOH as appropriate. Osmolarity was 300–330 mOsmol.

Recordings were made using either an Axopatch 1D amplifier (Axon Instruments, Union City, CA, USA) or a HEKA EPC 9 amplifier using Pulse acquisition and analysis software (HEKA). Currents were filtered at 3–5 kHz, sampled at 20–100 KHz, and recorded on hard disk for later analysis. Series resistance ranged from 2–7 MOhm and was compensated by 80% in all experiments. An approximate value of whole cell capacitance was determined by nulling the amplifier capacitance compensation circuit (Axoptach 1D) or automatically by EPC 9. Leak current was subtracted on line using a P/8 protocol. Cells were exposed to drugs via a series of flow pipes positioned about 200 μM from the cells. Fast application of ATP, capsaicin and acid were made with valve controlled sewer pipes.

### Data analysis

Significant differences between means were tested using unpaired, two tailed Students *t*-test as noted. All data are expressed as mean ± S.E.mean unless otherwise indicated.

### Drugs and Chemicals

DAMGO ([Tyr-D-Ala-Gly-MePhe-Gly-ol]enkephalin), U-69593 ((+)-(5-, 7-, 8-)-*N*-methyl-*N*-[7-(1-pyrrolidinyl)-1-oxaspiro[4.5]dec-8-yl]benzeneacetamide), deltorphin II, capsaicin, laminin, polylysine were from Sigma. Buffer salts were from Sigma. F-12, L-15 and fetal bovine serum were from GIBCO. Tetrodotoxin and human NT-3 were from Alomone Laboratories. NGF (mouse 2.5 S) was from either Upstate or Sigma. Papain and collagenase were from Worthington Biochemical Corporation (Freehold, NJ, USA), dispase was from Roche Applied Sciences.

## Competing interests

MC, LAN & EWM declare that they have no conflicts of interest relating to this work.

## Authors' contributions

MC performed experiments and wrote the draft of the paper, LAN performed experiments and EWM conceived the study. All the authors were involved in the experimental design, data analysis and writing the final paper. All the authors have all seen and approved the final paper.
